# BH-CIFOL: Case-Intensional First Order Logic (II) Branching Histories

**DOI:** 10.1007/s10992-013-9292-4

**Published:** 2013-08-25

**Authors:** Nuel Belnap, Thomas Müller

**Affiliations:** 1 Department of Philosophy, University of Pittsburgh, 1001 Cathedral of Learning, Pittsburgh, PA 15260 USA; 2 Department of Philosophy, Utrecht University, Janskerkhof 13a, 3512 BL Utrecht, The Netherlands

**Keywords:** Modal logic, Quantification, Sortal, Tracing, Substance, Identity, Indeterminism, Branching

## Abstract

This paper follows Part I of our essay on case-intensional first-order logic (CIFOL; Belnap and Müller (2013)). We introduce a framework of branching histories to take account of indeterminism. Our system BH-CIFOL adds structure to the cases, which in Part I formed just a set: a case in BH-CIFOL is a moment/history pair, specifying both an element of a partial ordering of moments and one of the total courses of events (extending all the way into the future) that that moment is part of. This framework allows us to define the familiar Ockhamist temporal/modal connectives, most notably for past, future, and settledness. The novelty of our framework becomes visible in our discussion of substances in branching histories, i.e., in its first-order part. That discussion shows how the basic idea of tracing an individual thing from case to case via an absolute property is applicable in a branching histories framework. We stress the importance of keeping apart extensionality and moment-definiteness, and give a formal account of how the specification of natural sortals and natural qualities turns out to be a coordination task in BH-CIFOL. We also provide a detailed answer to Lewis’s well-known argument against branching histories, exposing the fallacy in that argument.

## Introduction to this Part

This is Part II of a two-part essay. In Part I [4] we introduced CIFOL, a “case-intensional first-order logic,” as a general purpose quantified modal logic derived from Bressan [6]. Here we refine and enrich CIFOL to take account of indeterminism, relying for that purpose on a branching structure that we call “branching histories”; we will call the resulting system BH-CIFOL.^1^ Much, however, remains exactly the same as in Part I.

### BH-CIFOL Grammar

We rehearse material from Section 2.1 of Part I for ease of reference. The principal “parts of speech” in BH-CIFOL are terms, sentences, operators, and predicates, all defined by recursion on complexity, and certain connectives. Among the atomic constants there are sentential constants, *p*, predicate constants, *P*, individual constants, *c*, and operator constants, *f *. Among the atomic terms, there is also a set *Vars* of individual variables, with *x, y, z* ranging over them, and there is a special individual constant, *, to figure as a sign of non-existence. Individual terms, with *α, β* ranging over them, arise by applying an *n*-ary operator (either constant or *λ*-operator), *η*, to an *n*-tuple of terms: *η* (*α*_1_, . . . , *α*_*n*_). There is a distinguished two-place predicate constant for use in case-dependent identity sentences: *α*_1_= *α*_2_.^2^ Using $\Theta $ to range over predicates, additional sentences come by applying an *n*-ary predicate (either constant or $\lambda $-predicate), $\Theta $, to an *n*-tuple of terms: $\Theta (\alpha _{1},\ldots ,\alpha _n)$. Sentences arise from these via the usual truth-functional connectives such as negation, conjunction, disjunction, and the conditional and biconditional: $\neg , \wedge , \vee , \rightarrow , \leftrightarrow $; the modal connectives $\square $ and $\lozenge $ for necessity and possibility; and the usual first-order quantifiers, $\exists x$ and $\forall x$, applied to sentences. $\Phi $ ranges over sentences. Occasionally we use $\phi $ to stand for a particular sentence.

CIFOL features unrestricted formation of *ι*-terms (definite descriptions) *ι* $(\Phi )$, $\lambda $-predicates $\lambda x(\Phi )$, and $\lambda $-operators $\lambda x (\alpha )$.^3^ A definite description is an individual term. Applying a $\lambda $-operator, $\lambda x (\alpha )$, to a term, $\beta $, issues in a term, $(\lambda x (\alpha ))\beta $. Applying a $\lambda $-predicate, $\lambda x(\Phi )$, to a term, $\beta $, issues in a sentence, $(\lambda x(\Phi ))\beta $. A $\lambda $-operator [$\lambda $-predicate] may occur only in an operator [predicate] position (on pain of ascending past the first order). Finally, an expression, whether open or closed, is either an operator, $\eta $, or a predicate, $\Theta $, or an individual term, $\alpha $, or a sentence, $\Phi $; and in the latter two cases is categorematic. We let $\xi $ range over expressions. To aid reading, in Section 4 we distinguish predicates, $\Theta $, into two important kinds, absolute “sortal” predicates, $\Sigma $, and extensional “quality” predicates, $\Xi $, in terms of certain properties; but neither CIFOL nor BH-CIFOL makes an official syntactic distinction between sortals and qualities.

### BH-CIFOL Parameters and Models

The fundamental BH-CIFOL semantic parameters are summed up in a “BH-CIFOL model,” $\mathcal {M}$.^4^ These parameters remain fixed in the course of inductive semantic evaluation. In addition, an assignment, $\delta $, of (intensional) values to free variables is required in order to evaluate expressions containing free variables. Finally, extensions require an additional parameter, the “case,” since truth values and all other extensions are case-relative. (We explain the required notion of a case for BH-CIFOL via Definition 2 below.) Thus, evaluating intensions requires a model $\mathcal {M}$ together with an assignment $\delta $, and evaluating extensions requires in addition a case.^5^

#### **Definition 1** (Model)

A BH-CIFOL model, $ \mathcal {M}$, is an ordered list,

1 $$ \mathcal{M} = \langle M, \le, T, m_{C}, D, \mathcal{I}\rangle, $$

where (1) *M* is the set of “moments,” i.e. world-spanning momentary events, (2) $\le $ is a partial order on *M*, (3) *T* is the set of “times,” (4) $m_{C} \in M$ is the “moment of utterance,” (5) *D* is a domain of possible “extensions,” and (6) $\mathcal {I}$ is a function assigning an appropriate “intension” to each (atomic) constant: $\mathcal {I}(c)\in (\Gamma \mapsto D)$, $\mathcal {I}(p)\in (\Gamma \mapsto \mathbf {2})$, $\mathcal {I}(P)\in (\Gamma \mapsto ((\Gamma \mapsto D)\mapsto \mathbf {2}))$, and $\mathcal {I}(f)\in (\Gamma \mapsto ((\Gamma \ \mapsto D)\mapsto D))$. In the clause for (6), we are using $\Gamma $ to stand for the set of all cases $M/H$ according to Definition 2 below. Furthermore, $\langle M,\le \rangle $ and *T* are subject to the constraints of Postulates 1–3 (see Section 2.1 below).

In CIFOL, cases were entirely unstructured, whereas, as we soon explain, each case in BH-CIFOL has an intrinsic structure. Nevertheless, an intension in BH-CIFOL is, as in CIFOL, always a function from the set of cases into extensions. That is, using $(X\mapsto Y)$ as the set of functions from *X* into *Y* each intension belongs to $(\Gamma \mapsto Y)$, for suitable *Y*.

BH-CIFOL retains CIFOL’s general method of extensions and intensions: Every categorematic expression $\xi $ (term or sentence, open or closed) has an extension, $ext_{\mathcal {M},\delta , \gamma }(\xi )$, in each model, $\mathcal {M}$, assignment $\delta $, and case $\gamma $, and an intension, $int_\mathcal{M} \delta (\xi )$, which is a function from cases to appropriate extensions. As for CIFOL, the general link between extensions and intension is given by the following:

2 $$ ext_{\mathcal{M}, \delta, \gamma}(\xi) = (int_{{\mathcal{M}},\delta}(\xi))(\gamma); \quad\quad int_{{\mathcal{M}},\delta}( \xi) = \lambda\gamma (ext_{{\mathcal{M}}, \delta, \gamma} (\xi)). $$

Many parameters in $\mathcal {M}$ are used only for certain expressions; for example, $m_{C}$ is used only for moment-of-utterance dependent expressions (indexicals). Also, $\delta $ is used only for expressions with free variables.

Given a model, $\mathcal {M}$, truth of a sentence $\Phi $ in a case $\gamma $ is expressed by

3 $$ ext_{{\mathcal{M}}, \delta, \gamma}(\Phi) = \textbf{T}, $$

or, alternatively, via the truth predicate,

4 $$ \mathcal{M}, \delta, \gamma \models \Phi. $$

## Metaphysics of Branching Histories

In this section we lay out what must be included for branching histories; and first of all we set out the metaphysical underpinnings from which we can construct a proper set of cases and the domain, *D*.

In our development of BH-CIFOL, we will preserve CIFOL’s logical virtues of easiness of use, uniformity, and power. We will add to the usefulness of the system by means of a more specific analysis of the notions of sortal vs. qualitative properties, and thereby, of an individual thing (a substance) and its properties.

Let us hasten to stress that by providing an analysis of cases in the framework of branching histories, we are not claiming to give the ultimate analysis of possibility and necessity, or of modality *de re*. If, contrary to fact, we were to make any claims as to giving “the one true modal logic,” the claim might be that that is what CIFOL provides, generally speaking. BH-CIFOL, on the other hand, gives a useful, more fine-grained analysis of one specific kind of modality: the type of real, temporal-modal possibility and necessity that we have to deal with in the actual world, and which informs and constrains our agency. By stressing the importance of real possibility, we do not want to diminish the usefulness of other types of modality for specific aims—for example, there seem to be *some* good uses of thought experiments in science and in philosophy (Rawls’s idea of an original position comes to mind), and the modality involved in them, which is meant to give us access to conceptual knowledge, does not have to be of the kind we are presently tackling. Nor is it part of our claim that other types of modality can in any useful sense be reduced to what we are dealing with here.

### Our World, Moments, Histories, Cases, Times

We mentioned in Part I that the logical framework of CIFOL leaves the interpretation of the set of cases completely open. Two main examples for cases we mentioned were, first, linear time (a case is a moment or perhaps an interval), and second, cases that represent possibilities without taking into account the passing of time. It is quite natural to want to combine the two. One way, which goes by the name of “$T \times W$,” is to combine time and modality by forming the Cartesian product of a linearly ordered set of times, $\langle T, \leq \rangle $, and an unordered set of possibilities (perhaps “possible worlds”), *W *. The cases with which we end up on this approach are pairs $\langle t,w\rangle $, with $t\in T$ and $w\in W$. There are good reasons to favor a different approach (see Belnap et al. [5], Ch.7A.6). That approach, due to Kripke, Prior and Thomason, went by the name of “branching time”.^6^ *Moments* are the fundamental entities in this representation of Our World. Be warned, however: The moments are not yet the cases, nor are they times. Each moment—we use the parameter, *M*, to denote the set of all of them—is to be construed as a world-wide (possible) instantaneous *event*.^7^ To represent indeterminism, we introduce the parameter $\le $ as a tree-like (partial) causal order on *M*:

#### **Postulate 1** (Our World)

Our World is a nonempty partial order $\langle M,\le \rangle $ such that there is no backward branching (for all $x,y,z$, if $x\leq z$ and $y\leq z$, then ($x\leq y$ or $y\leq x$)).

We define < as the strict mate of $\le $: $m_{1} < m_2 \leftrightarrow _{df} m_{1}\le m_{2}$ but $m_{1}\neq m_{2}$. Because of branching, we need to read $m_1<m_{2}$ with care in either of two equivalent ways: either as “$m_{1}$ is in the causal past of $m_{2}$” or as “$m_{2}$ is in the future of possibilities of $m_{1}$”. We let *m* range over *M*.

A critical defined notion is that of a *history*, a linear course of momentary events that may well stretch without limit both forward and backward:

#### **Definition 2** (Histories and cases)

A set $h\subseteq M$ is a *history* iff *h* is a maximal chain in *M* (that is, any two elements of *h* are comparable via $\leq $, and no proper superset of *h* has that property). We write *H* for the set of all histories, *h* for an arbitrary member of *H*, and $H_{(m)}$ for the set of histories containing the moment $m\in M$.

A *moment/history* pair is an ordered pair $\langle m, h\rangle $ with $m\in M$ and $h\in H$ such that $m\in h$; we write $m/h$ for a moment-history pair, presupposing that $m\in h$.

A *case* in BH-CIFOL is defined as a moment/history pair. We let $M/H$ be the set of all cases.

One (optional) way to think about an $m/h$ pair is to think of it as a kind of “temporal-modal vector,” with *m* a starting position and *h* a kind of direction amid future possibilities. The moment and history parameters evidently fail to be independent: It makes no sense to refer to a pair $\langle m, h\rangle $ as a case, unless $m\in h$. More prosaically, both *m* and *h* are used in evaluating tenses; for example, whether, in a given case, Caroline will arrive on time depends on what happens in case the future of *m* unfolds according to history *h*. Having exposed the constitution of cases in BH-CIFOL, we henceforth write

5 $$ ext_{{\mathcal{M}}, \delta, m/h}(\xi) = (int_{{\mathcal{M}},\delta}(\xi))(m/h);\ int_{{\mathcal{M}},\delta}( \xi) = \lambda (m/h) (ext_{{\mathcal{M}}, \delta, m/h} (\xi)) $$

in place of (2), and

6 $$ \mathcal{M}, \delta, m/h \models \Phi $$

in place of (4).

Since histories are to represent really possible courses of events, we suppose that none are entirely isolated:

#### **Postulate 2** (Histories intersect)

Each two distinct histories intersect, and their intersection has always a last moment (a splitting point).

To say that histories $h_{1}, h_{2}$ split at a moment, *m*, is equally to say that the pair branches at *m*. Either way, it is plainly histories (courses of events) that branch among themselves rather than times. We do, however, also require as a parameter a linearly ordered set, *T*, of *times*, which certainly will not branch. The parameter *T* is needed below for the $\mathit {Now:\/}$ and the $\mathit {AT}{_{\,n}}$ operators.^8^

#### **Postulate 3** (Times)

*T* is a partition of *M* such that (1) for any $\tau \in T$ and any history *h*, the set $\tau \cap h$ has exactly one member and (2) *T* respects the ordering, that is, for $h,h'$ histories and $\tau _{1},\tau _{2}\in T$, we have $h\cap \tau _{1} \leq h\cap \tau _{2}$ iff $h'\cap \tau _{1} \leq h'\cap \tau _{2}$.^9^ On *T*, we have an induced ordering, $\leq _{T}$ (we set $\tau _{1}\leq _{T} \tau _{2}$ iff there are $m_{1}\in \tau _{1}$ and $m_{2}\in \tau _{2}$ such that $m_{1}\leq m_{2}$; condition (2) above assures well-definedness).

Though not essential for understanding indeterminism, we often assume that *T* (and therefore each history) is order-isomorphic to the positive and negative reals, so that time has no first nor last moment.

Just as *H* represents the set of alternative histories (that is, courses of events) *in which* moments occur, so *T* represents the set of times *at which* moments occur. It is clear from these postulates and definitions that in spite of the hold of the phrase “branching time” in philosophical logic, times do not branch: It is only histories that branch one from another.

We shall need to refer to each of *M*, *H*, and *T* in giving semantic explanations; we shall, however, exercise our option to treat them in different ways: *M* and *T* are intensional parameters, whereas *H* is defined in terms of *M*.

### From Branching Histories to BH-CIFOL

The choice to take cases to be moment/history pairs in branching histories is motivated by the idea that real cases for us—the cases that are behind real possibilities and necessities—are both temporal and modal. We are facing a future of open possibilities. That choice of $M/H$ as the set of all cases reflects back in two ways on the general logical framework of BH-CIFOL.

First, following the development of temporal logic, it is natural to extend the language by characterizing new temporal and modal connectives that make accessible the extra structure of our set of cases, $M/H$. These will be the temporal connectives *Was:* and *Will:*, and the so-called historical modality of settled truth, *Sett:*, as well as their duals. (We avoid the locutions “historical modality,” “historical necessity,” and “historical possibility” as too misleading; see note 11 below.) These and a few other connectives will be discussed in Section 3.

Second, our choice of $M/H$ has consequences for our analysis of qualitative and sortal properties that we adumbrated in, respectively, Sections 4.2 and 4.3 of Part I, as well as for the notion of a “thing.” We stressed in Part I that CIFOL invites the idea that members of the extensional domain *D* should not be thought of as things, contrary to the customary image of quantified modal logic as a theory of “possible worlds and their inhabitants.” The elements of *D* are *extensions*, fit to exist in a single case. We pointed out that in a temporal reading, it may be useful to think of the extensions as “stages,” but emphatically not in the sense of stages that are themselves individual things.^10^ This idea of extensions different from things means that an additional way of tracing a thing from case to case must be supplied, since the “rigid designation” idea of equality of extension across cases is not wanted in CIFOL, and a discourse-relative notion of counterpart seems too loose to ground an objective notion of tracing. As we spelled out in Part I, absolute properties in CIFOL supply the needed tracing principles. Such properties are both modally constant (if they apply to an individual intension in one case, they apply in all cases) and modally separated (no two different individual intensions falling under the property can have the same nonempty extension in any case). Absolute properties allow one to identify a whole individual intension (a map from cases to extensions) via a single nonempty extension in a single case (see our definition of $\bar {\alpha }_{\Theta }$ at the end of Section 4 of Part I).

The definition of an absolute property is purely formal, and provably there are many—indeed, too many. It would be uninformative to try and define an individual *thing* as an individual intension that can be traced under some absolute property—provably, for every individual intension we can define such a property, so that we should have to conclude that every individual intension represents a thing. It is however intuitively clear that some individual intensions (patterns of extensions across cases) correspond to proper things, while others are just some gerrymandered messes. Thus, despite grammatical appearances, it would be wrong to think that “Carlotta’s favorite thing,” which varies over time, identifies the individual intension of a proper thing, referring, as the case may be, to a cat, a book, a cuddly toy, or a bunch of flowers. Given what our world is like, there just aren’t any things that persist in such a strange way.

We stressed that in CIFOL speaking generally, admitting individual intensions that do not correspond to things is not a bug but a feature: It is not for logic, but for science and metaphysics to tell us what the things around us are, or which of our terms have thing-intensions and which don’t, or which of the many absolute properties are natural and which aren’t. In general, there is nothing more we, as logicians, can say. Consequently, in Part I we gave the following gloss on what a thing (an individual, or a substance) could be: “To be an individual in the concrete world is to be the value of a variable ranging over individual intensions that fall under some natural sortal (fn 28).”

In opting for temporal/modal cases in branching histories, we are adding metaphysical detail on the side of the cases, so that one may expect some metaphysical payoff on the side of things, or of natural sortals. That is indeed the case; we will give the details in Section 4.

It may be useful if we offer three preliminary comments on the metaphysical language that we are employing, keeping in mind that BH-CIFOL is a formal language that is intended to be useful in sorting out some elementary features of both our scientific and our everyday language concerning persistence from time to time and from case to case. (1) We try to use the following words and phrases as more or less interchangeable parts of our informal metaphysical and scientific language: *thing* or *proper thing*, *(concrete) individual, substance*. Plants and animals and persons are things, as well as ordinary *natural* inanimate objects. (We are not concerned with artifacts.) (2) Things fall under at least one informal sortal. BH-CIFOL provides a formal account of *sort*, or *sortal concept*, and a companion formal account of *quality*, much like the notions of a CIFOL-quality and a CIFOL-sortal in Part I, Defs. 14 and 19. (3) We follow Bressan and Part I in using *natural*, e.g. “natural sortal,” to suggest informally necessary and sufficient conditions. (It is we think hopeless to try to characterize “natural” in formal terms.) We claim as Theses that the formal notions of BH-CIFOL-sortal and BH-CIFOL-quality give necessary conditions for being a natural sortal, or a natural quality.

## Temporal and Modal Connectives

The branching histories framework affords the introduction of a number of new temporal and modal connectives. We introduce them in Sections 3.2–3.5.

### Grammar Common to this Part and Part I

The language treated or used in this part properly includes that considered in Part I: *E* (existence, Part I, Def. 1, see Definition 6 below), interpretation of individual, sentential, predicate, and function constants, $c, p, P, f$, extensions and intensions of sentences and terms, $\lambda $ constructions, truth-functional, modal, and quantificational connectives, unique existence and definite descriptions, defined predicates, and truth and validity. Each and every semantic clause for this common grammar, remains unchanged, except for replacing the Part I case parameter, $\gamma $, by a parameter, $m/h$, for moment-history pairs, and replacing $\Gamma $ with $M/H$ for the set of all cases. We exhibit just two of these systematically adapted clauses: The basic alethic modal connectives, “possibly” ($\lozenge $) and its dual, “necessarily” ($\square $), are straightforward **S5** modalities quantifying over the set of cases, $M/H$. Since we have defined the cases as $m/h$ pairs, the accounts of necessity and possibility now become the following (in our semantic clauses we use the truth-predicate form of Eq. 4 above, which is the briefest and most familiar form of Def. 11 of Part I).

$$ \mathcal{M},\delta, m/h \models \square\Phi \leftrightarrow_{df} \forall (m^{\prime}/h^{\prime}) [\mathcal{M},\delta,\/m^{\prime}/h^{\prime} \models \Phi]. $$

$$ \mathcal{M},\delta, m/h \models \lozenge\Phi \leftrightarrow_{df} \exists (m^{\prime}/h^{\prime}) [\mathcal{M},\delta,m^{\prime}/h^{\prime} \models \Phi]. $$

#### Convention.

When every constant in $\Phi $ lies in the scope of $\square $ or $\lozenge $, $m/h$ is evidently an irrelevant parameter and so may be dropped. Similarly, $\delta $ may be dropped as a parameter for every expression not containing any unbound variable.

### Grammar Special to this Part

For the modal connectives *Sett:* (“settled true” means “true no matter what happens in the future”), 11 *Will:* (the future modality, Prior’s *F*) and *Was:* (past, Prior’s *P*), we need to unpack the structure of the set of cases as a set of moment/history pairs; the clauses are the standard Ockhamist ones (see Thomason [21, 22]):^12^

$$ \mathcal{M},\delta, m/h \models {Sett:\, \/}\Phi \leftrightarrow_{df} \forall h^{\prime}[\mbox{if}\ m\in h^{\prime} \,\, \text{then} \, \,\mathcal{M},\delta, m/h^{\prime} \models \Phi]. $$

$$ \mathcal{M}, \delta, m/h \models {Will:\,\/}\Phi \leftrightarrow_{df} \exists m'\in h\ \mbox{such that}\ m<m'\ \text{and}\, \, \mathcal{M},\delta, m^{\prime}/h \models \Phi. $$

$$ \mathcal{M}, \delta, m/h \models {Was:\, \/}\Phi \leftrightarrow_{df} \exists m'\in h\ \mbox{such that}\, \, m'<m\ \text{and}\ \mathcal{M},\delta, m^{\prime}/h \models \Phi. $$

The dual connectives are also often useful, we can introduce them as abbreviations:^13^

$${Poss:\,\/}\Phi \Leftrightarrow_{df} \neg {Sett: \, \/}\neg\Phi; $$

$${Will-always:\, \/}\Phi \Leftrightarrow_{df} \neg {Will:\,\/}\neg\Phi; $$

$${Was-always:\, \/}\Phi \Leftrightarrow_{df} \neg {Was:\, \/}\neg\Phi. $$

Given that we are working in a no-backward-branching, connected structure, we could have defined the global **S5** necessity connective explicitly in our language. In fact, if histories have no first nor last moments (e.g., if they are order-isomorphic to the real number line), we have the following rather simple validity:^14^

$$\square\Phi \leftrightarrow {Was-always: \, \/}{Sett:\, \/}{Will-always: \,\/}{Sett:\, \/}\Phi. $$

To see this, from left to right, assume that the right hand side of the biconditional is false at some $m/h$:

$$ \mathcal{M}, \delta,m/h\not\models {Was-always: \, \/}{Sett:\,\/}{Will-always: \, \/}{Sett: \,\/}\Phi. $$

Since all the operators correspond to universal quantification over moments or histories, this must be due to some

$$ \mathcal{M}, \delta,m'/h'\not\models\Phi. $$

By the semantic clause for $\square $, this implies

$$ \mathcal{M}, \delta,m/h\not\models\square \Phi. $$

In the other direction, assume that the left hand side is false at some $m/h$ as witnessed by $m'/h'$. We leave it as an exercise for the reader to prove that the suite of operators on the right hand side allows one to reach that $m'/h'$ from $m/h$ (Postulate 2 from Section 2.1 above will be needed).

We can also define a simple notion of “always,” which stays local to the current history of evaluation:

$${Always:\,\/}\Phi \Leftrightarrow_{df} \Phi\,\,\wedge\,\,{Was-always:\,\/}\Phi \,\,\wedge\,\,{Will-always:\, \/}\Phi. $$

### *Now*:

Hans Kamp first pointed out that in linear tense logic, *Now*: gives access to the time of utterance.^15^ The adaptation to branching histories is straightforward: Instead of the time of utterance, we take as a parameter the very moment of utterance, $m_{C}$. That is, the semantics of *Now*: refers to the moment of utterance, $m_{C}$, which is by Definition 1 an element of the model, $\mathcal {M}$, details concerning which we postpone to Section 3.4. That moment gives rise to a time of the utterance, $t_{C}$ (defined as the unique $t_{C}\in T$ for which $m_{C}\in t_{C}$). The clause for *Now*: is therefore

$$ \mathcal{M}, \delta, m/h \models {Now: \, \/} \Phi\ \leftrightarrow_{df}\ {\mathcal{M}}, \delta, m'/h \models\Phi\ \mbox{for the (unique)}\ m'\in h\cap t_C. $$

Note that “*Now*:” doesn’t necessarily get us back to the *moment* of utterance; it will only guarantee to get us back to the *time* of utterance, on the present history of evaluation. This is presumably how “now” works in English; [5], 246, gives the example “I’m not now rich, but if the coin had landed heads I would now be rich,” showing that “now” in English allows for inconsistent “nows.” In BH-CIFOL, the corresponding formal fact is that many sentences of the form

$${Now:\,\/}\neg\Phi \,\,\wedge\,\, {Was:\/}{Poss:\, \/}{Now:\,\/}\Phi $$

are satisfiable.

### *Actually*:, Moment of Utterance, and Worlds

We are indebted to David Kaplan [11] and David Lewis [12] for emphasizing that actuality in the context of multiple possibilities is best taken indexically.^16^ As Kaplan puts it, we refer to actuality with a new semantic parameter, the context of utterance of a specimen sentence to be subject to semantic analysis. According to Def. 1, we include the moment of utterance, $m_{C}$, as a parameter in the model, $\mathcal {M}$, omitting for present purposes such other elements of context as speaker, listener, place, and so on. By including $m_{C}$ in $\mathcal {M}$, Def. 1 together with Eqs. 5 and 6 of Section 2.1 in effect postulate that $m_{C}$ is a parameter of each of extension, intension, and true-at. The general interrelation between the extension operator, the intension operator, and the truth predicate, mentioned at the end of Section 1, stays in place.^17^

Lewis’s indexical analysis of “actually” simply refers to an “actual world” given by the context. In the general framework of case-intensional semantics, this transfers to using an *actual case*, to be specified by the context of utterance. In the current branching histories framework, however, there is no such thing as “the actual case,” which would have to be “the actual moment/history pair” singled out by the context. It is unproblematic to identify the *moment* of the purported actual moment/history pair as the moment, $m_{C}$, of utterance. The insuperable difficulty comes with making sense, in an indeterministic setting, of “*the* history containing the moment of utterance.” Given that moments typically belong to multiple histories, and that histories extend into the future beyond the dissolution of our solar system, with unending open possibilities, there is no (actual rather than pretend) uniqueness to be had. There is no such thing as “the actual history.” We therefore settle for the following reading:^18^What is *actually* true will be defined as whatever is settled true at the moment $m_{C}$:

$${\mathcal{M}}, \delta, m/h \models \textit{Actually: \/}\Phi\ \leftrightarrow_{df} {\mathcal{M}}, \delta, m_C/h' \models \Phi\ \mbox{for all}\ h'\in H_{(m_C)}. $$

Speaking ontologically, from among the moments in *M* it is only $m_{C}$ and the moments in its past that are actual. Other moments either are possible or were possible, always speaking in relation to $m_{C}$. Using the $\textit {AT}\ensuremath {_{\,n}}$ modality to be introduced in the following section, BH-CIFOL allows us to express the thought that on January 1, 2012 (at time $t_{n}\in T$), it could have been sunny ($\phi $) but it actually wasn’t:

$$Was: Poss: AT_{n}\phi \,\,\wedge\,\,Actually: AT_{n}\neg \phi. $$

Note that for us as for Lewis, “real” is non-relational, in contrast with “actual.” “Real” just means “in the structure,” so we are modal realists in that sense: There are real possibilities. It is, however, worthwhile to draw a basic contrast between BH-CIFOL metaphysics vs. possible-worlds metaphysics: Lewis-Kaplan worlds do not have any parts in common, whereas histories overlap. But what about Our World? What makes it a world? We fully endorse Lewis’s characterization: “if two things are spatiotemporally related, then they are worldmates,” and we also follow Lewis in “more or less” adopting the converse ([12], 71). It follows that the assemblage of all histories constitutes, via overlap, a single world, Our World. Since Our World contains all real possibilities, the metaphysical aspect of our investigation needs no others.^19^ We do not, however, subscribe to the idea of disjoint possible worlds existing in some peculiar modal space. Because histories pair-wise intersect, our real possibilities are linked spatio-temporally, as Lewis wisely requires of worldmates.

### At a Time

It is also useful to have a connective to be read “at time *t*.” Belnap et al. [5] let *t* be a term to be evaluated like any other. This turns *AT* into what Curry called a “mixed nector,” to be completed by a term, *t*, and a sentence, $\Phi $:

$${\mathcal{M}}, \delta, m/h \models AT_{t}\Phi, $$

to be read “At time *t*, $\Phi $.” That would seem to be the way to go in order to give a truly uniform treatment. The proper implementation of this strategy would require, however, that one see to it that *t* falls under a natural sortal for times by specifying intensions and extensions of time-terms, probably via an account of real numbers. (Bressan [6] has such an account, but it takes us well beyond the first order.) For illustrative purposes, we avoid these complexities and follow the simple treatment of the $\textit {AT}\ensuremath {_{\,n}}$ connective introduced in Part I, Section 5.2: We posit a recursive enumeration, $t_{0}, \ldots , t_{n}, \ldots $ (possibly finite) of some sufficiently large and interesting subset of *T* that we may take as “nameable times,” where we may think of each *n* as a name for $t_{n}$. We suppose that for each *n* there is a primitive connective $\textit {AT}\ensuremath {_{\,n}}$, with the following semantics:^20^

$$ \mathcal{M}, \delta, m/h \models AT\ensuremath{_{\,n}} \Phi\ \leftrightarrow_{df}\ \mathcal{M}, \delta, m'/h \models\Phi\ \mathrm{for \, the \, (unique)}\ m'\in h\cap t_n. $$

Then, the sentence $AT\ensuremath {_{\,n}}\Phi $, to be read “At time $t_{n}, \Phi $,” is true at index $\ensuremath \mathcal {M}, \delta , m/h$ iff $\Phi $ is true at that moment $m'$ in history *h* that occurs at time $t_{n}$.

The example from Section 3.4 illustrates the use of $AT\ensuremath {_{\,n}}$. That example shows that even though indeterminism in a branching-histories setting is tied to the notion of an open future of possibilities, we can express indeterminism independently of the indexical *Will*:. In fact we can express indeterminism, in the sense of incompatible options for the same date, without any indexicals (i.e., without temporal operators, *Now*:, or *Actually*:): Let $t_{k}$ be the date of the sea battle at Salamis, $t_{j}$ the date of the day before, and let $\phi $ stand for “a sea battle is taking place.” Then we can express the indeterminacy of what happened at that fateful day as follows:

$$ AT{_{j}} [ \lozenge{AT}\ensuremath{_{k}}\phi \,\,\wedge\,\,\lozenge{AT}\ensuremath{_{k}}\neg\phi ]. $$

## Substances in Branching Histories

We stressed in Section 2.2 that the specific choice of moment/history pairs as cases not only allows for the introduction of new connectives, as we saw in Section 3, but also has repercussions for the discussion of things and their properties. The main ideas we will be dealing with are due to the internal structure of the set of cases. Cases $m/h$ and $m/h'$, for $h,h'\in H_{(m)}$ and $h\neq h'$, are certainly different—but they are not, as it were, as different as cases $m/h$ and $m'/h'$ belonging to different moments. What distinguishes $m/h$ and $m/h'$ is not what is present, but only what will be in the future. This triggers the thought that whether a thing is present in a case or not (which is surely a local matter), should only depend on the *m*-part of the $m/h$-case. It may also suggest that at least for ordinary properties, whether a thing has the property or not, should be independent of the *h* component of the case as well. We will follow both these thoughts in Section 4.1, to arrive, in Section 4.2, at partial explications of a sortal property, a quality, and a thing, that are designed to be fine-grained versions of the general CIFOL explications.^21^

### Moment-Definiteness

We said in Part I that the definitions of extensionality, absoluteness, and so on, are also part of the core of CIFOL (although, to repeat, not a “creative” part). These general CIFOL definitions give us a handle on two metaphysically important notions: that of a quality, which holds or doesn’t hold of a thing depending only on what that thing is like in a single case, and of a sortal property, under which a thing can be traced from case to case. The definitions are fully general and do not depend on the specific nature of the cases. In BH-CIFOL we build upon these definitions by adding specific machinery to connect cases belonging to the same moment.

The fact that in BH-CIFOL cases are moment/history pairs rather than just moments, gives rise to tensions already at the propositional level: In defining a model providing an interpretation of the propositional language of branching histories, should the assignment of truth values to atomic sentences be a function from the moments, or from moment/history pairs, to truth values? Thomason [21] opts for the latter, for the sake of uniformity of substitution. The other option is however also popular, and can be defended on philosophical grounds:^22^Why should the truth value of an atomic sentence, which in temporal logic is present tensed, be affected by what happens next? This argument can in turn be countered by pointing out that it is not so clear what atomic sentences are (apart from verum and falsum, perhaps, for which the issue, however, doesn’t arise)—sentences have internal structure, and there is no reason *a priori* why among those sentences that we pick out as atomic, there shouldn’t be some whose truth-value does depend on what happens next (even though they are present-tensed). Perhaps “Peter chooses Mary” is like that. Our general point, however, is that from the point of view of language-design at the highest level, it seems unnecessarily confining to enter a once-for-all prohibition against endowing a grammatically atomic sentence with a complex semantics.

CIFOL’s semantics assigns a propositional intension (a function from the set of cases, $\Gamma $, to the set of truth-values, **2**) to sentence constants. In BH-CIFOL, where $\Gamma $ is replaced by $M/H$, this amounts to the more fine-grained choice from among the two discussed above: The semantics allows that an atomic sentence, *p*, is true at $m/h$ and false at $m/h'$. That is however not the end of the matter—we can, if we want, spell out explicitly the option that the truth value of a sentence, $\Phi $, should depend at most on the moment, and thereby express the more coarse-grained semantics for atomic *p*. We can use the modality, ${Sett\hspace *{-3pt}:\/}$, to help. We don’t want to say that $\Phi $ is settled true (${Sett\hspace *{-3pt}:\/} \, \Phi $), but only that it is settled one way or the other. That is, we want a modality that expresses that it is settled whether or not $\Phi $:

#### **Definition 3** (Settled-whether)

$${SettWh:\/} \Phi \leftrightarrow_{df} ({Sett:\/} \Phi \,\,\vee\,\, {Sett:\/} \neg \Phi).$$

If we wished to force all atomic sentences to be settled one way or the other (which we do not), we could use Def. 3 to introduce the corresponding axiom schema, ${SettWh\hspace *{-3pt}:\/} p$.

As we said, depending on what the atomic sentences are, the option of settled excluded middle may be warranted or not. It would certainly seem to be warranted for purely qualitative atomic sentences, such as “it is raining,” or “it is sunny.” What about non-atomic sentences such as simple predications, $\Theta \alpha $? That depends. In order to have a handle on this question, we give a definition of “moment-definite property” along the present lines:

#### **Definition 4** (Moment-definite)

A property $\Theta $ is *moment-definite at* $m/h$ iff:

$$ \mathcal{M},\delta, m/h\models \forall x\,{SettWh: \, \/} \Theta x. $$

A property $\Theta $ is *moment-definite* iff it is moment-definite in every case, i.e., iff the following holds:

$$ \mathcal{M},\delta \models \square \forall x\, {SettWh: \,\/} \Theta x. $$

Which properties are moment-definite? One guiding idea is that whether something has a purely qualitative property or not, cannot depend on what will happen in the future. Thus, we certainly cannot expect moment-definiteness for properties that contain an implicit reference to what will happen, such as being mortally wounded. But it seems that we can expect moment-definiteness of ordinary qualitative properties, such as being red, or weighing 65 kg.

In Part I we characterized qualitative properties, for which we will use $\Xi $, as *extensional*, i.e., case-bound (a quality should hold or not, depending only on what is so in a single case):

$$ \mathcal{M},\delta \models \square \forall x\forall y [x=y\rightarrow (\Xi x\leftrightarrow \Xi y)]. $$

Moment-definiteness and extensionality can thus be motivated by similar considerations—but they don’t go together well and can only be combined in a trivial way, as the following fact shows. For convenience of expression, we use the fact that the extension $ext_{\ensuremath \mathcal {M},\delta ,m/h}(\Theta )$ of a predicate $\Theta $ at a case $m/h$, which is defined to be a function from individual intensions $\bar {z}\in (M/H \mapsto D)$ to the set of truth values $\{\textbf {T},\textbf {F}\}$, can also be viewed as the characteristic function of the set of intensions falling under the predicate in that case. We define this set as the “extension-prime” of the predicate:

$$ext^{\prime}_{ \mathcal{M}, \delta,m/h}(\Theta) =_{df} \{\bar{z} \mid \bar{z}\in (M/H \mapsto D)\,\,\wedge\,\, (ext_{ \mathcal{M},\delta,m/h}(\Theta))(\bar{z}) =\textbf{T}\}. $$

#### **Fact 1**

Let $\Theta $ be both extensional and moment-definite, and let $m/h$, $m/h'$, with $h\neq h'$, be two different cases belonging to the same moment. Then $\Theta $ either applies to no individual intension at $m/h$, or to all of them. That is, $ext^{\prime }_{\ensuremath \mathcal {M},\delta ,m/h}(\Theta ) =\emptyset $ or $ext^{\prime }_{\ensuremath \mathcal {M},\delta ,m/h}(\Theta ) = (M/H\mapsto D)$.

#### Proof

Assume otherwise, so that there are individual intensions $\bar {x},\bar {y}\in (M/H\mapsto D)$ such that $\bar {x}\in ext^{\prime }_{\ensuremath \mathcal {M},\delta , m/h} (\Theta )$ and $\bar {y}\not \in ext^{\prime }_{\ensuremath \mathcal {M},\delta ,m/h} (\Theta )$. By moment-definiteness, this carries over to case $m/h'$, i.e., we also have $\bar {x}\in ext^{\prime }_{\ensuremath \mathcal {M},\delta ,m/h'}(\Theta )$ and $\bar {y}\not \in ext^{\prime }_{\ensuremath \mathcal {M},\delta ,m/h'}(\Theta )$. Now let the individual intension $\bar {z}\in (M/H\mapsto D)$ be defined as follows:

$$\bar{z}(m''/h'') =_{df} \left\{ \begin{array}{ll} \bar{x}(m''/h'') & \mathrm{i{f}f}\ m''\neq m\,\,\text{or}\,\,h''\neq h;\\ \bar{y}(m''/h'') & \mathrm{i{f}f}\ m''=m\,\,\text{and}\,\,h''=h. \end{array} \right. $$

By extensionality (note that $\bar {z}(m/h') = \bar {x}(m/h')$), we have $\bar {z}\in ext^{\prime }_{\ensuremath \mathcal {M},\delta ,m/h'}(\Theta )$, and so by moment-definiteness, we also have $\bar {z}\in ext^{\prime }_{\ensuremath \mathcal {M},\delta ,m/h }(\Theta )$. But then, by extensionality (note that $\bar {z}(m/h) = \bar {y}(m/h)$), we also have $\bar {y}\in ext^{\prime }_{\ensuremath \mathcal {M},\delta ,m/h}(\Theta )$, contradicting our assumption. □

How does it happen that the two similarly motivated notions come apart so easily? We offer two diagnoses. First, consider the following formal point: An extensional property depends on one single case. Moment-definiteness, however, looks at more than one case at a time—given that the moment in question admits more than one possible future. This is hidden by the fact that we can express moment-definiteness as “depends on only one moment,” similar to glossing extensionality as “depends on only one case.” In BH-CIFOL, cases are more fine-grained than moments, so the two notions come apart.

Second, there is a problem with the implicit reading of “something” as “some proper thing,” which we relied on in order to argue for the moment-definiteness of qualitative properties. BH-CIFOL variables, however, range over all individual intensions, including gerrymandered ones—and for them, it is not reasonable to expect moment-definiteness even of natural qualities.

The upshot is that we are facing a task of coordinating our natural qualities and our natural kinds; we cannot tackle them in isolation. A similar point has been made in connection with induction and lawlike statements: As Davidson [8] argues, the fact that Goodman’s “grue” (green if examined before *t* and blue otherwise) is not a property that we can use for induction, depends not on the appearance of a time index in its definition, but rather on the fact that among the things around us there are emeralds and sapphires, but no emerires (which are emeralds before *t* and sapphires thereafter). In the world around us, there are no things that persist in that strange way. But that’s an empirical fact, for science to discover, not an *a priori* truth—and thus, a proper logic should allow us to reason about emerires. BH-CIFOL does.

In order to express the coordination between natural qualities and natural kinds, we introduce the following definition. Although the definition doesn’t use the formal concepts “extensional” and “absolute,” the principal application is to an extensional property $\Xi $, with $\Sigma $ an absolute property—which is why we use the convention laid down at the end of Section 1.1.

#### **Definition 5** (Moment-definite for …)

A property $\Xi $ is *moment-definite for* $\Sigma $ *s iff*

$$ \mathcal{M},\delta\models \square\forall x\,[\Sigma x\rightarrow {SettWh: \,\/} \Xi x]. $$

We illustrate by an example that is similar to our horse stories from Section 5 of Part I, and which brings out the specific issues connected with moment-definiteness; see Tables 1, 2, 3, 4. In our tables we use the same conventions as in Section 5 of Part I: The columns specify the extension-prime of the respective predicate in the given case, that is, they list all the intensions (functions from cases to extensions) falling under the predicate in that case. A “-” means that *any* extension from *D* can occupy the respective position.

**Table 1:** Horses (modally constant)

Horse \ Case	$m_0/h_{1}$	$m_0/h_{2}$	$m_1/h_{1}$	$m_2/h_{2}$
Andy	abcd	abcd	abcd	abcd
Doris	ef**	ef**	ef**	ef**
Jack	jkln	jkln	jkln	jkln

**Table 2:** Existing horses (moment-definite)

Existing horse \ Case	$m_0/h_{1}$	$m_0/h_{2}$	$m_1/h_{1}$	$m_2/h_{2}$
Andy	abcd	abcd	abcd	abcd
Doris	ef**	ef**		
Jack	jkln	jkln	jkln	jkln

**Table 3:** Colors, and winning

Property \ Case	$m_0/h_{1}$	$m_0/h_{2}$	$m_1/h_{1}$	$m_2/h_{2}$
Black	a- - -	-b- -	- -c-	- - -d
	e- - -	-f- -		
Brown	j- - -	-k- -	- -l-	- - -n
Wins	∅	∅	- -c-	- - -n
Horse that will win	abcd	jkln	∅	∅
Brack	a- - -	-k- -	- -c-	- - -n

**Table 4:** Truth values of color attributions

The horse that will win is \ Case	$m_0/h_{1}$	$m_0/h_{2}$	$m_1/h_{1}$	$m_2/h_{2}$
Black	**T**	**F**	**F**	**F**
Brown	**F**	**T**	**F**	**F**
Brack	**T**	**T**	**F**	**F**

**Fig. 1:**
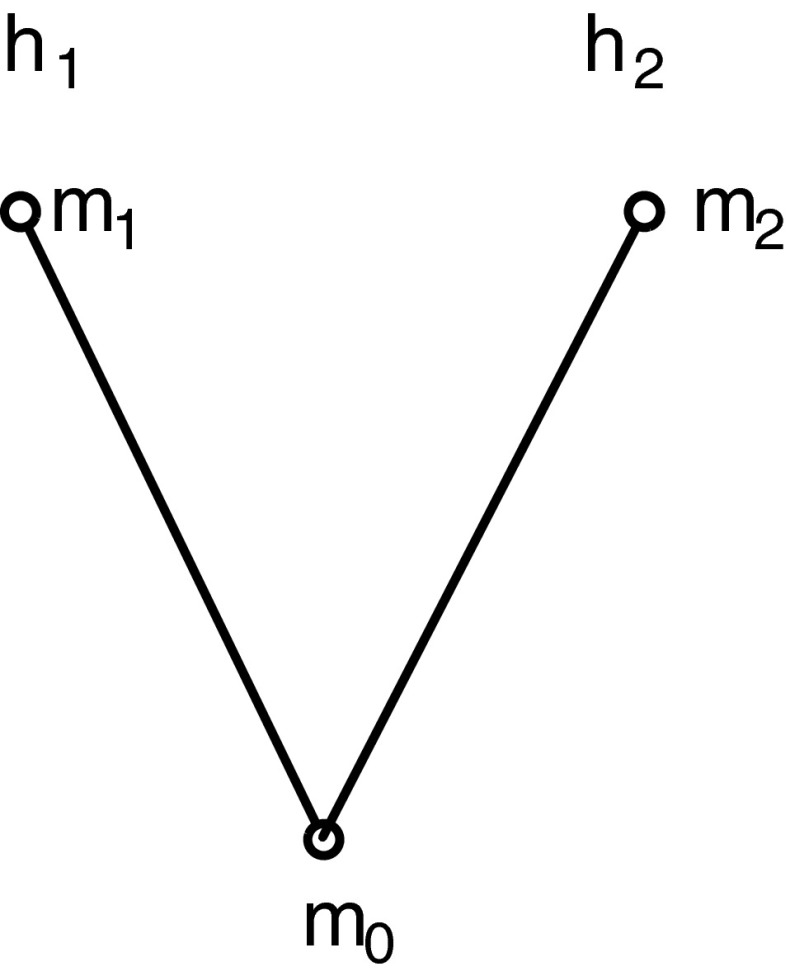
A branching model with three moments $m_{0}$, $m_{1}$, and $m_{2}$, two histories $h_1=\{m_{0},m_{1}\}$ and $h_2=\{m_{0},m_{2}\}$, and four moment/history cases $\gamma _1=m_0/h_{1}$, $\gamma _2=m_0/h_{2}$, $\gamma _3=m_1/h_{1}$, and $\gamma _4=m_2/h_{2}$

In the following examples, we work with the simplest non-trivial branching structure, which has three moments $m_{0}$, $m_{1}$, and $m_{2}$, such that $m_0<m_{1}$ and $m_0<m_{2}$, whereas $m_{1}$ and $m_{2}$ are incomparable (see Fig. 1). This gives rise to two histories, $h_{1} = \{m_{0},m_{1}\}$ and $h_{2} = \{m_{0},m_{2}\}$. Accordingly, the branching model contains *four* cases: $M/H = \{\gamma _{1} =_{df} m_0/h_{1}$, $\gamma _{2} =_{df} m_0/h_{2}$, $\gamma _{3} =_{df} m_1/h_{1}$, and $\gamma _{4} =_{df} m_2/h_{2}\}$. The domain *D* contains at least the same horse-extensions as in Part I, $\{$a,b,c,d,e,f,g,h,i,j,k,l,n,*$\}$.

In the following tables, important properties of predicates manifest themselves as follows: Modal constancy (Part I, Def. 16) means that all columns have the same entries: if an intension falls under the predicate in one case, it falls under it in all cases (see, for example, Table 1). Moment-definiteness (Def. 4) means that all columns belonging to the same moment have the same entries (as in Tables 1 and 2). Modal separation (Part I, Def. 17) means that in any column, no two different intensions have the same extension in any case, except, perhaps, for * (see, for example, Table 1). Absoluteness (Part I, Def. 18) is the conjunction of modal constancy and modal separation, as exemplified by Table 1. Finally, extensionality means that whether an intension occurs in a column, can only depend on the extension for the respective case. Thus, for example, if the column for case $m_0/h_{2}$ contains the intension jb*g, as for “Black” in Table 3, then any intension $\bar {z}$ with $\bar {z}(m_0/h_2)=\mbox {b}$ has to be listed in that column as well. As you can see, the “-” notation used in Table 3 is specifically designed to notate extensional predicates.

Note first that while modal constancy implies moment-definiteness, being (everywhere) moment-definite is not the same as being modally constant; it is a more local affair. A comparison of Tables 1 and 2 illustrates this: While “horse” is modally constant (in fact, absolute), “existing horse” is moment-definite, but it is not modally constant, as the second line in Table 2 shows.

Table 3 illustrates the issue of coordination between things and qualities. The properties of being black, brown, or being a winner, are extensional—and thus, since they are *not* trivial, they are not moment-definite. Being black and being brown *are*, however, moment-definite *for horses* (Def. 5), as can be checked by inspection. The qualities of being black or brown, and the absolute property or being a horse, are made for one another, as it were. On the other hand, we know that being brown cannot be moment-definite with respect to all intensions, and the table illustrates this as well. From the property “Horse that will win” one can form the definite description “the horse that will win,” which by the BH-CIFOL rules (Part I, Def. 10) has the intension ak**. As one can read off from Table 4, it is neither settled true that the horse that will win is brown, nor that it is not brown, nor that it is black, nor that it is not black.

And there’s nothing wrong with that: Clearly, which color the horse that will win has at moment $m_{0}$, depends on which horse will win, and that is still open at $m_{0}$—Andy will win on $h_{1}$, and Jack on $h_{2}$. If we want a quality that goes together with a gerrymandered intension like that of “the horse that will win,” we can try “brack” from Table 3, which is our addition to the palette of gerrymandered color-terms like “grue,” “bleen,” or “gred.” Note however that while these more familiar examples apply gerrymandering with respect to cases in linear time, what is going on in our example is more specific, and less familiar: The gerrymandering is with respect to cases belonging to the same moment, and thus, *a fortiori*, with respect to the same time.

So it would seem that the question which are the concrete individuals (things, substances), and which are the proper natural qualities, is chiefly a coordination task. This is almost completely so, but there is one quality that should be settled as a matter of logic, viz., existence. In Part I, we defined an existence predicate, *E*, whose definition in BH-CIFOL remains unchanged from Def. 1:

#### **Definition 6** (Existence predicate)

$$\square\forall x [Ex \leftrightarrow_{df} x\neq *]. $$

Existence of $\alpha $ at case $m/h$ is then expressed by

$$ \mathcal{M}, \delta, m/h \models E\alpha. $$

Since existence is thereby extensional, and is not trivial, we know by Fact 1 that it cannot be moment-definite. But we can argue, as a piece of substantial yet perhaps uncontroversial metaphysics, that existence *of concrete individuals* should be everywhere settled-whether. This notion also transfers into a property of properties. We define:

#### **Definition 7** (Existence-settled)

An individual $\alpha $ is *existence-settled* iff

$$ \mathcal{M}, \delta \models \square ({SettWh:\/}\, E\alpha). $$

A property $\Theta $ is *existence-settled* iff

$$ \mathcal{M}, \delta \models \square\forall x\, [\Theta x\rightarrow ({SettWh:\/}\, Ex)]. $$

Our claim is thus that substances are existence-settled: Whether *some thing* exists, cannot depend on what will happen next. Whether *something* exists, on the other hand—for example, the winner of tomorrow’s race—may depend on how things play out later (e.g., the race may be canceled, and there will be no winner).

### Things and Sorts

Many metaphysicians have remarked that “thing,” while grammatically a sortal, shows behavior untypical of sortals. It is, for example, unclear how we can individuate, let alone count, things. One useful diagnosis of the behavior of “thing” in English is that it often functions as a pro-common-noun ([9], 34), picking up a common noun from the context.

From the point of view of BH-CIFOL, we can offer a framework for “thing,” and thereby, the notion of a substance, building on the definitions of Section 4.1. Combining Def. 7 with the notion of absoluteness, we arrive at BH-CIFOL’s necessary condition on sortal terms. That condition is more fine-grained than the criterion of absoluteness, which was what CIFOL alone had to offer. We retain from Part I the policy of splitting our claims about sortals and qualities into purely formal definitions giving necessary and sufficient formal conditions (see Part I, Defs. 14 and 19), and substantive theses giving only necessary conditions for the natural notions (see Part I, Thesis 1 and 2).

#### **Definition 8** (BH-CIFOL sortal)

A property $\Sigma $ is a *BH-CIFOL sortal* $\leftrightarrow _{df}$ $\Sigma $ is absolute and existence-settled.

One might be momentarily tempted to add “existence-implying” (Def. 12 from Part I) until one recalls that we want to treat *Man* as absolute without supposing that the *Man*, Socrates, exists in every case.^23^

#### **Thesis 1** (Sortals)

Up to an approximation, every natural sortal is a BH-CIFOL sortal.

This criterion takes into account the intuition that whether a proper thing (an individual intension falling under a natural sortal) exists in a case or not, should not depend on what the future will bring. Existence-or-non-existence is a settled matter. We mention another formally specifiable condition that could be added as a further necessary condition for being a natural sortal: uninterrupted existence for substances. Certainly a biological individual cannot cease to exist and afterwards begin to exist again. This can be spelled out as follows:

#### **Definition 9** (Uninterrupted existence)

An individual $\alpha $ has *uninterrupted existence* iff

$$ \mathcal{M},\delta \models \square (E\alpha \rightarrow \neg{Will:\, \/} (\neg E\alpha\,\,\wedge\,\, {Will:\, \/} E\alpha)). $$

A property $\Theta $ *secures uninterrupted existence* iff

$$ \mathcal{M}, \delta \models \square\forall x\, [\Theta x\rightarrow (E\alpha \rightarrow \neg{Will: \,\/} (\neg E\alpha\,\,\wedge\,\, {Will: \,\/} E\alpha))]. $$

No matter whether uninterrupted existence is added as an additional condition or not, it is, however, clear that the definition of a BH-CIFOL sortal cannot provide a sufficient condition for being a natural sortal. There are still far too many BH-CIFOL sortals around. In fact, for any individual intension that is existence-settled, there is a BH-CIFOL sortal under which it falls. Thus, given, for example, that we know that “cat” and “dog” are natural sortals, so that cat- and dog-intensions are existence-settled, we can take two such intensions existing in the same cases and mix them to arrive at an intension that is also existence-settled, but which is, for example, equal to the dog in case in rains tomorrow and equal to the cat in case it doesn’t rain tomorrow. *Given that we know that cats and dogs are substances*, such a gerrymandered intension just cannot be a substance as well—but there is a BH-CIFOL sortal under which it falls.

Let us try to spell out this thought in more detail, and see what we can do with it. In a world (like ours) in which cats and dogs are substances, it would be strange if there were a substance-term mixing these *per moment*. An individual falling under such a sortal would be a dog at some moments and a cat at others. For all we know, no living being persists in such a way—but still, that’s a broadly empirical issue. (Grafted plants probably show some aspects of such behavior.) The gerrymandered intensions we considered in the previous paragraph are however much worse: Their gerrymandering is not one of persisting by switching sorts from moment to moment, but one of having, at one and the same moment, an extension belonging to different sorts depending on what the future will bring. It is a substantive, but rather plausible thesis that in a world of cats and dogs, there can be no such substances as well.

In giving this explication, our point is not that we can have no use for an intension wildly mixing a cat and a dog. Maybe it corresponds to “the animal that will win at tomorrow’s pet show.” The point is just that it is a plausible thesis to claim that we cannot have such gerrymandered individual intensions falling under a substance sortal alongside with cats and dogs. We also stress that our point is not to defend that metaphysical thesis (even though, just for the record, we believe it to be rather plausible). What we are looking for is a means of expressing it and thus, to open the room for a rigorous discussion.

How to express this thesis? Here is one seductive way to approach it, which we will however reject because it it involves metalinguistic resources in a way contrary to the spirit of (BH-)CIFOL. Think of the extension of a substance-term $\alpha $ (a singular term falling under a natural substance sortal) in a case $m/h$ as a momentary stage of the substance. What the future will bring can make no difference to the fact that at the moment *m*, that stage is present. So, it seems natural to demand that that stage be present at all cases involving the moment, *m*:

$$\forall h'\in H_{(m)}\, [ext_{ \mathcal{M},\delta,m/h'}(\alpha) = ext_{ \mathcal{M},\delta,m/h}(\alpha)]. $$

This demand secures that the extension of the term is rigid across cases belonging to the same moment. We reject this approach to spelling out our thesis because it involves comparing extensions across cases, which is not in the spirit of BH-CIFOL. But then how can we express the thesis? A little reflection shows that there is no hope to express the thesis via a monadic property of properties. But we can, similarly to the coordination between sorts and qualities discussed in the previous section, express the thesis as a coordination principle between different substance properties:

#### **Definition 10** (Harmony)

Two BH-CIFOL sortals $\Sigma _{1}$ and $\Sigma _{2}$ are *harmonious* iff

$$ \mathcal{M},\delta \models \square\forall x\,\forall y\, [(\Sigma_{1} x\,\,\wedge\,\, \Sigma_2 y)\rightarrow {SettWh: \, \/} x=y]. $$

A class of BH-CIFOL sortals is *harmonious* iff any two of its members are.

So here is a thesis expressing an interface to science and metaphysics:

#### **Thesis 2**

The class of natural sortals is harmonious.

This thesis rules out that our gerrymandered “winner of tomorrow’s pet show” falls under a natural sortal along with cats and dogs. We can now venture to express what a thing is: Something (some individual intension) is a thing iff it falls under some natural sortal. This is best expressed disjunctively, via an axiom that incorporates those sortal predicates that, under a given interpretation and with respect to a given application, are to be natural sortals. In a world of only cats and dogs, that axiom would look like this:^24^

$$ \mathcal{M} \models \square\forall x [Thing(x)\leftrightarrow (Dog(x)\,\,\vee\,\, Cat(x))]. $$

Thesis 2 then amounts to the substantial thesis that “thing,” while not a proper sortal, behaves at least locally like a sortal. On the assumption of that thesis, we can prove the following fact about settledness of the identity of things:

#### **Fact 2**

Let the property “thing” be defined disjunctively, via the axiom

$$ \mathcal{M} \models \square\forall x [Thing(x) \leftrightarrow (\Sigma_1x\,\,\vee\,\,\ldots\,\,\vee\,\, \Sigma_nx)] $$

for a harmonious class $\{ \Sigma _{i}\mid 1\leq i\leq n\}$ of BH-CIFOL sortal properties (*n* some natural number). Then we have

$$ \mathcal{M}\models \square\forall x\forall y\,[(Thing(x)\,\,\wedge\,\, Thing(y))\rightarrow {SettWh: \, \/} x=y]. $$

#### Proof

Let the assumption hold, let $\alpha $, $\beta $ be given and let $m/h\in M/H$ be a case for which we have

$$ \mathcal{M}, m/h\models Thing(\alpha) \,\,\wedge\,\, Thing(\beta). $$

Then there must be $\Sigma _{i}$ and $\Sigma _{j}$, $1\leq i,j\leq n$, for which

$$ \mathcal{M}, m/h\models \Sigma_{i}\alpha\,\,\wedge\,\, \Sigma_{j}\beta. $$

The claim then follows by the definition of harmony (Def. 10). □

In our discussion of qualities above we motivated the idea that a quality can be appropriate for some sort of things, in the sense that for these things, the quality behaves as we expect it to: It applies or doesn’t, given just a single case, and whether it applies to a thing, does not depend on what the future will bring. We will now put this idea to work and show the usefulness of our thesis of harmony.

For the record, here is our definition of BH-CIFOL qualities and the corresponding thesis about natural qualities. Unlike our discussion of sortals, we cannot add any new general conditions here, so that definition and thesis stay as in Part I:

#### **Definition 11** (BH-CIFOL quality)

A property, $\Xi $, is a *BH-CIFOL quality* $\leftrightarrow _{df}$ $\Xi $ is extensional (Part I, Def. 13).

#### **Thesis 3** (Qualities)

Up to an idealization, natural qualities are BH-CIFOL qualities.

We think of this Thesis as analogous to Theses 1 and 2: it gives the necessary conditions, statable in pure BH-CIFOL, for a property to be a quality. The following little fact shows that BH-CIFOL qualities and harmonious BH-CIFOL sortals interact fruitfully: Moment-definiteness of a BH-CIFOL quality transfers from one BH-CIFOL sortal to another, provided that they are harmonious and overlap.

#### **Fact 3**

Let $\Sigma _{1}$, $\Sigma _{2}$ be natural sortals (so that $\Sigma _{1}$ and $\Sigma _{2}$ are harmonious), and let $\Xi $ be a BH-CIFOL quality that is moment-definite for $\Sigma _{1}$. If we have

$$ \mathcal{M} \models \square\forall x\, [\Sigma_2x \rightarrow \exists y\,[\Sigma_{1} y\,\,\wedge\,\, x=y]] $$

then $\Xi $ is moment-definite for $\Sigma _{2}$ as well.

#### Proof

Let $m/h\in M/H$ and $\alpha $ be given such that

$$ \mathcal{M}, m/h\models \Sigma_{2}\alpha. $$

Our assumption of overlap gives us

$$ \mathcal{M},m/h\models \exists y [\Sigma_1y \,\,\wedge\,\, \alpha=y]. $$

Call the witness (which, by the way, is unique by modal separation of $\Sigma _{1}$) $\beta $. By harmony between $\Sigma _{1}$ and $\Sigma _{2}$, we have

$$ \mathcal{M}, m/h\models {Sett: \, \/} \alpha=\beta, $$

and the fact that $\Xi $ is moment-definite for $\Sigma _{1}$, gives us

$$ \mathcal{M}, m/h \models {SettWh: \,\/} \Xi\alpha. $$

Via the settled identity of $\beta $ and $\alpha $ and extensionality of $\Xi $, we thus also have

$$ \mathcal{M}, m/h \models {SettWh: \,\/} \Xi\beta. $$

□

Logically speaking, this is all. Let us, however, give a metaphysical gloss on this fact. When would the overlap assumption between $\Sigma _{1}$ and $\Sigma _{2}$ be satisfied? We may think of $\Sigma _{1}$ as standing for “parcel of matter” and $\Sigma _{2}$ for some biological sort, for example, “rabbit.” The overlap assumption then says that in every case, for any rabbit there is a parcel of matter that is, *in that particluar case*, identical with the rabbit. (Thus, if in a given case the rabbit-term has a non-empty extension—if there is, as we may want to say, a rabbit-stage present—then there is a parcel of matter-term that has the same extension in that case; and if the rabbit-term has the empty extension, then there is a parcel of matter-term that has the empty extension as well.) It is clear from rudimentary biology that the rabbit and the matter are traced differently: The rabbit has a metabolism, and constantly exchanges matter with its environment while alive; so while the rabbit is fairly easy to trace, its matter very quickly disperses—minimally, with each breath it takes. Fact 3 then allows us to transfer good qualitative properties of the matter, such as mass, to the rabbit itself. This way of viewing the difficult issue of constitution of biological entities by physical ones may not suit everybody. Note, however, that it does not amount to any form of physicalism that would threaten the usefulness or autonomy of the so-called special sciences such as biology. Constitution as *case-relative* identity is much less controversial than the thesis of constitution as identity discussed in contemporary analytical metaphysics. In fact, we have so far not found any convincing counterexample.

### Individuals and Branching

We hope to have made plausible, by means of some examples and a few formal results, that BH-CIFOL offers a rich and formally detailed account of sorts, qualities and things that can provide the background for a formally perspicuous discussion of a number of metaphysical theses and scientific arguments. We do not embark on any metaphysical discussions in this essay, which is meant to remain within the bounds of (subject-neutral) logic, just spelling out the formal interface that such metaphysical discussion could employ.

There is, however, one issue that will occur to many readers, and which we feel needs commenting: Many metaphysicians are convinced that a branching representation of possibilities is ultimately incoherent, and thus useless. With respect to our framework, this would mean that while CIFOL, as a general logic, would not be affected, the system of BH-CIFOL presented here would need to be abandoned, perhaps in favor of a “$T\times W$” representation of temporal/modal possibilities as mentioned in Section 2.1. While it may be that a number of our results and arguments have formal analogues in that framework, the $M/H$ based account given here would need to be rejected.

What’s the trouble? Numerous places in the literature as well as many discussions with colleagues show that an argument against branching due to Lewis [12] has been highly influential. Lewis’s argument goes as follows (p. 207f.; “divergence” is Lewis’s expression for his specific $T\times W$-like approach):

The trouble with branching exactly is that it conflicts with our ordinary presupposition that we have a single future. If two futures are equally mine, one with a sea fight tomorrow and one without, it is nonsense to wonder which way it will be—it will be both ways—and yet I do wonder. The theory of branching suits those who think this wondering is nonsense. Or those who think the wondering makes sense only if reconstrued: you have leave to wonder about the sea fight, provided that really you wonder not about what tomorrow will bring but about what today predetermines. But a modal realist who thinks in the ordinary way that it makes sense to wonder about what the future will bring, and who distinguishes this from wondering about what is already predetermined, will reject branching in favour of divergence.

This argument invokes a lot of resources; the epistemic notion of wondering plays a crucial role. We have not provided a semantics for any epistemic operators so far, and we will not give a comprehensive picture. Still, let us try to relate the argument to our framework.^25^

For illustration, we stick to the branching structure of Fig. 1 in Section 4.1 with three moments $\{m_{0},m_{1},m_{2}\}$, two histories $h_1=\{m_{0},m_{1}\}$, $h_2=\{m_{0},m_{2}\}$, two times, $t_{0} = \{m_{0}\}$, $t_1=\{m_{1},m_{2}\}$, four cases, $M/H = \{m_0/h_{1}$, $m_0/h_{2}$, $m_1/h_{1}$, $m_2/h_{2}\}$, and with a domain including $*$ and the horse-extensions from Part I together with some “man” extensions. Let us introduce a further individual term, “Rick,” for Rick, Andy’s jockey, with intension pqrs, and a sortal, “man”, such that the individual intension pqrs, and nothing else, falls under “man” in each of the four cases. Thereby, “man” is a BH-CIFOL sortal, as you can check; and we know that it is in fact a natural sortal. Since Lewis’s example invokes first-personal thought, we also need, minimally, an indexical expression, “I,” and a “speaker of the context” parameter (an individual intension) $\bar {s}$ in our model, $ \mathcal {M}$, such that the intension of “I” is $\bar {s}$.^26^ Instead of the sea fight, let us stick to the horse race example and consider the sentence, “Andy wins at $t_{1}$,” symbolized as $\phi = {AT}\ensuremath {_{\,1}} Wins(Andy)$.^27^ This sentence, as we see from the semantics of “at a time” (Section 3.5) and from Table 3 (Section 4.1), depends for its truth only on the history: It is true in cases $m_0/h_{1}$ and $m_1/h_{1}$, and false in the other cases, $m_0/h_{2}$ and $m_2/h_{2}$. Note that by the semantics of “at a time,” we have that

$$ \mathcal{M}, m/h\models \neg{AT}\ensuremath{_{\,1}} Wins(Andy) \leftrightarrow {AT}\ensuremath{_{\,1}}\neg Wins(Andy), $$

so that “$\neg \phi $” allows both readings, “it is not the case that at $t_{1}$, Andy wins” and “at $t_{1}$, Andy does not win.” As a last piece of set-up, let us assume that, for any $\Phi $, one cannot know whether $\Phi $ (in the usual factive sense of “to know”) unless it is settled whether $\Phi $, and let us also agree that wondering whether $\Phi $ implies that one does not know whether $\Phi $.

Let us look at what Rick has to say at the moment of the context of utterance, $m_{C} = m_{0}$.

- “Two futures are equally mine, one losing and one winning.”While it is perhaps a bit odd to say this,^28^ it is clear enough what is meant, and true when properly understood: Both futures are equally Rick’s, in the sense that both are equally possible. Formally,
  $${Poss:\/}\phi \,\,\wedge\,\, {Poss:\/}\neg\phi, $$
  and by the semantics of the ${Poss\hspace *{-3pt}: \,\/}$ operator, we also have
  $${Sett: \,\/} ({Poss: \, \/}\phi \,\,\wedge\,\, {Poss:\,\/}\neg\phi). $$
  Thus we can grant that Rick can say that “I know that two futures are equally mine”; formally,
  $$Know_{I}\,({Poss: \,\/}\phi \,\,\wedge\,\, {Poss: \,\/}\neg\phi). $$
- “I wonder which way it will be.”The best we can do with this, given our limited resources, is to follow the implication mentioned above, and render this as “I do not know which way it will be,”
  $$\neg Know_{I} \phi \,\,\wedge\,\, \neg Know_{I} \neg\phi. $$
  Strictly speaking, we cannot go any further from here (your not knowing something is compatible with much else either being the case or not being the case), but let us strengthen to “I cannot know which way it will be” (which is perhaps implicated at least weakly by “I wonder”), and allow ourselves to read the above link between knowledge and settledness backwards, so that we arrive at “It is not settled which way it will be,”
  $$\neg {SettWh: \,\/}\phi. $$
  This is true (even settled true) at the moment of utterance, and so Rick can know this.
- “It will be both ways.”This is simply false if understood as something that Rick says, i.e., as “It will be that both, I will win and I will not win” (at the same time, viz., $t_{1}$). No moment can witness a contradiction, so that we have
  $${Sett: \,\/} \neg (\phi\,\,\wedge\,\,\neg\phi), $$
  and again, this is a settled truth that Rick can know.
  Thus, Lewis’s claim that “it is nonsense to wonder which way it will be” on a branching conception, which depends on the attribution to branching theorists of the claim that “it will be both ways,” is unsupported, and his discussion of the reconstrued content of the wondering, which is offered as the only way out for the branching theorist, is beside the point.

To wrap up, on the branching histories picture that we are offering, at the moment of utterance, $m_C=m_{0}$, it is not settled whether Andy (and thereby Rick) will win or lose at $t_{1}$, because it is both possible that he will win, and possible that he will lose, and thus it makes sense for Rick to wonder which way it will be. Whatever the outcome, it certainly won’t be both ways. It’s as simple as that.

How is it, then, that Lewis’s argument continues to convince so may philosophers? While this is strictly speaking a psychological question to be answered per individual, we offer the following general diagnosis. In his argument, Lewis appeals to two different perspectives on the scenario at hand, and mixes them in a confusing way. First, there is the internal perspective, the one from which a speaker situated at a specific moment utters a sentence or has a thought, such as “I wonder which way it will be.” Our (indexical) language is tied to this perspective, and the analysis of Rick’s utterances (or thoughts) above shows that this perspective, if followed consistently, is unproblematic.

Second, however, the fact that many people tend to see some truth in Lewis’s (on the face of it, outrageous) claim that “it will be both ways” seems to indicate that a shift of perspective is taking place in the quoted passage. From an external, God’s eye view on the branching structure, we see that Rick is represented as an individual intension that exists (has a non-empty extension) in all four cases, including those involving the incompatible moments, $m_{1}$ and $m_{2}$. At the time of the end of the race, $t_{1}$, there are two moments, one winning and one losing. So it may seem reasonable (but it isn’t) to say that “at $t_{1}$, Rick is both winning and losing.” One shouldn’t say this because it is needlessly confusing: It is much better to say that at $t_{1}$, there are two (incompatible) moments, $m_{1}$ and $m_{2}$, one of which is a winning moment for Rick and the other, a losing moment. The individual, Rick, has “incompatible properties at the same time, $t_{1}$,” but all this means, really, is that at that time there are incompatible moments $m_{1}$ and $m_{2}$, both in the future of possibilities of $m_{0}$, and these moments are indeed incompatible in the sense that at one of them, Rick is winning and at the other, he isn’t. These moments, with all their content, are indeed “equally real” from the God’s eye perspective, but this merely means that they are both really possible. Their representation, such as via our Fig. 1 in Section 4.1 of this essay, is actual, but this does not mean that both incompatible moments are actual. As we said in Section 3.4, following Lewis, “actual” functions indexically, and is thereby bound to the internal perspective.

If we properly distinguish these two perspectives, we see that Lewis’s error is to employ the internal, indexical language that Rick speaks to report a fact of the external perspective. Lewis says “It will be both ways,” thereby using the indexical future tense (“it will be”) tied to the internal perspective, but then switches to the external perspective from which one sees two incompatible moments at time $t_{1}$ (“both ways”). Consistently, Lewis should either stick to the internal perspective, from which Rick can truthfully say “It will be one way (but I don’t know which),” or employ external language in describing a feature of the branching structure, for example, “at time $t_{1}$, there are two incompatible moments both equally real” (i.e., really possible, being parts of the model). He can even say “it is both ways,” since on pain of contradiction that will clearly be understood as “one way at this place in the structure and another way at another place.” Branching theorists are usually quite acutely aware of the need to keep these perspectives—the object language and the metalanguage giving the semantics—strictly separate; in fact, the branching histories framework originates in careful semantic studies of tensed languages (see note 6 above). We are not aware of any literature propounding a branching histories framework in the tradition of Prior and Thomason in which it was claimed that “it will be both ways,” and Lewis has cited no evidence to the contrary.

In order to prove the usefulness of keeping the two perspectives separate, let us look at another thing Rick says (or thinks) according to Lewis:

- “I have a single future.”

This seems a reasonable enough thing to say, and we can give a sensible interpretation from the internal perspective, with the sentence uttered at $m_{0}$. It would seem appropriate to render this in the form “for any future time, exactly one of two incompatible things will happen.” Indeed, we can use the above example of winning or losing at $t_{1}$, which generalizes easily to other times and subject matters. The following holds at $m_{0}$:

$${Sett:\, \/} ({AT}\ensuremath{_{\,1}}Wins(Andy) \,\,\vee\,\, {AT}\ensuremath{_{\,1}}\neg Wins(Andy)).</p><p class="noindent">$$

That is, it is settled (and therefore, knowable) that at the future time $t_{1}$, with respect to winning or losing, things will be exactly one way or the other. This should of course not be confused with the following, which is false at $m_{0}$:

$${Sett: \,\/} {AT}\ensuremath{_{\,1}}Wins(Andy) \,\,\vee\,\, {Sett: \,\/} {AT}\ensuremath{_{\,1}}\neg Wins(Andy). $$

Given that in our model, there are two relevantly different possibilities for the future as of $m_{0}$, it is neither settled that at $t_{1}$, Andy will win, nor settled that at $t_{1}$, Andy will lose.

A switch of perspectives here leads to an interpretation on which the sentence “I have a single future” seems false—but again, the switch of perspective is not warranted. It is true that the model does not represent Rick as having only one possible history—he exists (has a non-empty extension) in two partially overlapping, but also partially incompatible histories, $h_{1}$ and $h_{2}$. Any indeterministic model contains incompatible histories—that is the mark of indeterminism in contrast to determinism. So only a deterministic (linear) model could guarantee that an individual, such as Rick, exists on just one history. Still, from the internal perspective of speaking, thinking and wondering, it is settled that the future won’t be “both ways.”

To sum up our somewhat lengthy discussion, we have shown that Lewis’s argument against branching pulls no weight, since it is based on a conflation of two distinct perspectives. BH-CIFOL helps to keep these perspectives separate. There is no problem about representing individuals in a branching histories framework.

## Conclusions

In concluding this second part of our essay, we hope to have achieved what we set out to do: to introduce case-intensional first order logic as a versatile and useful quantified modal logic. We laid out its general framework, CIFOL, in Part I, starting with a comparative discussion of its relation with other frameworks, its general grammar, and its general semantics, which features intensional variables, intensional predication, and extensional identity. To repeat, the main innovation of Bressan [6] was to propose this general framework, with neutral notions of “cases” and “extensions,” together with specific definitions providing an interface for the discussion of the tracing of individuals across cases; the key definition of Part I is Def. 17 of modal separation, the most innovative element in Bressan’s notion of an absolute property (Def. 18). We are still struck by the fact that this notion, which is far more versatile and useful than the common notions of rigid designation or counterpart theory, is not used much more widely. 29

In Part I we explained the notion of tracing and some of its uses in detail, using a hypothetical example involving a few horses. We did not in that part give any explicit structure to the cases, and indeed, different notions of a case may be used to fill in our examples from Part I. In this second part, we left the neutrality of the cases behind, thereby moving our discussion closer to the topic of metaphysics. To repeat, we do not believe that quantified modal logic is a topic where metaphysics meets logic in the sense that metaphysical claims should shape the logic. Too much of that, in our view, is happening in standard systems combining modality and quantification, in which metaphysical assumptions tend to support extensionalist leanings. It is, however, true that in quantified modal logic, metaphysical discussions do suggest themselves, since so many metaphysical notions can find an expression in that logical language. Our approach for Part II was to follow one metaphysical line of thought and see how indeterminism, the combination of time and real possibility, can be represented.

In this second part, we thus introduced cases as moment/history ($m/h$) pairs in a framework of branching histories, which posits a partial, backwards-linear ordering of moments and identifies histories, total possible courses of events, with maximal linear chains in the ordering. Times partition the set of moments, so that there can be different incompatible moments at the same time—different incompatible possibilities for the same time. A case, as an $m/h$ pair (with $m\in h$), identifies both a moment and one of the possible futures as of that moment. This approach is familiar from Ockhamist temporal logic in the tradition of Prior [17] and Thomason [21], but it is given a new twist here by combining the quantificational machinery of CIFOL with the idea of branching histories, thus issuing in BH-CIFOL. Having motivated the framework in Sections 1 and 2, we went on to introduce the standard temporal/modal connectives for past (*Was*:), future (*Will*:), and settledness (*Sett*:), as well as the indexicals *Now*: and *Actually*: and connectives for “at a time.” None of the creative semantic clauses for these propositional connectives pertains specifically to the first order. As in Part I, the interface for discussing things, sorts and qualities comes by way of non-creative definitions, which extend the general CIFOL definitions of that earlier part. In discussing substances in branching histories in Section 4, we pointed out the crucial role of moment-definiteness vs. extensionality for an understanding of qualities, and of the settledness of existence and identity. We stress again that in these discussions, we did not derive metaphysical conclusions from the logical framework, but rather showed how certain metaphysical theses can be expressed and discussed in a formally perspicuous way. None of our discussions led to tweaking the logic, or to suggesting that it should be tweaked—rather, we gave definitions which can be added in specific applications of the BH-CIFOL framework in case they are warranted by separate arguments, which could come from metaphysics, science, or some other pertinent subject matter.

We would like to end by making explicit our proposal for the metaphysical discussions of individuals, their sorts and their qualities in an indeterministic setting (such as in the world around us). It appears to be useful to stick to the general CIFOL idea that individual things are best represented not as extensions, but as individual intensions falling under natural absolute properties (sortals), so that a term standing for a thing has an extension in all cases (the empty extension, $*$, signalling non-existence). No assumptions about the nature of extensions need be made, and no comparison of extensions between different cases is called for. It may be useful to think of extensions as “stages,” but not in the metaphysically loaded sense of Stages as separate individual things that pervades the current persistence debate. 30 The tracing of an individual from case to case is effected by absolute properties, which allow for tracing by being modally constant and modally separated. Qualities of such individuals are extensional properties. So far, this is the standard CIFOL story (apart from the extra requirement of settled existence for things, Def. 7). The specific addendum of BH-CIFOL lies in the interrelation among different sortal (natural absolute) properties, and among sortals and qualities. Our discussion of moment-definiteness motivated the idea that different sortal properties should be harmonious, meaning that the identity or distinctness of two intensions falling under different sortal properties in a given case should be a settled matter. A similar notion of harmony seems called for to describe the coordination between sortal and qualitative properties, as we illustrated with our “the winner is brack” horse story; Fact 3 shows that harmonious sorts facilitate the sharing of qualities. Taken together, BH-CIFOL allows for a picture in which a holistic network of sortal and qualitative properties is established gradually, in the course of scientific investigations. Such a picture may indeed be useful for describing certain episodes in the history of science. 31

In our final Section 4.3 we discussed Lewis’s oft-quoted argument against the representation of indeterminism via branching histories. We showed that BH-CIFOL allows one to separate clearly the two perspectives that Lewis runs together in his argument: an internal perspective of indexical language use, and an external perspective for describing a semantical model. As we showed via a detailed analysis, there is no problem representing individuals and their wonderings about the future in our branching framework. We venture to claim, therefore, that BH-CIFOL provides a useful first-order logic for indeterminism.

## Footnotes

1 By historical accident, an assemblage of possible courses of events that branch indeterministically has been call a “branching time” structure by nearly all workers following after Prior, Kripke, and Thomason—including ourselves. For mathematical concerns, nothing much hangs on the choice between “branching histories” and “branching time”; we, however, intend our study, though formal, to contribute to the metaphysical enterprise, so that terminology matters. Since we never think of time itself as branching, we abandon the phrase “branching time” in favor of “branching histories”.

2 Even though it is hard to think of identity as *world* dependent, taking it as *case* dependent is natural: “The winner will be (identical to) Ralph in case it rains, but not in case the sun shines.”

3
$\lambda $-operators and -predicates in BH-CIFOL are all one-place. One may simulate the use of binary $\lambda $-operators, for instance, with $\lambda x_{1}(\lambda x_{2}(\alpha )\beta _{2} )\beta _{1} $.

4 In this part, we reuse “$\mathcal {M}$,” which in Part I ranged over CIFOL models, to range over BH-CIFOL models.

5 In Part I we let $\gamma $ be our case variable everywhere, but after Definition 2 we will replace “$\gamma $” by “$m/h$” to reflect the detailed inner structure of a single case in BH-CIFOL. Correspondingly, we will then use “$M/H$” instead of “$\Gamma $” for the set of all cases.

6 For the early history of branching in temporal logic, see Ploug and Øhrstrøm [16].

7 There are other event structures that are invoked in representing the “open future” via branching histories; one is the “branching space-times” of Belnap [1], for which see also [3].

8 Note that postulating the existence of times constrains the admissible structures for moments, since all histories need to be order-isomorphic.

9 Here we are using the obvious induced ordering on singletons of moments: $\{m\}\leq \{m'\}$ iff $m\leq m'$.

10 To some it may also be attractive to think of the extensions as (sets of) tropes; on that interpretation, it is immediate that the extensions are not themselves things.

11 “Settled truth” is a better phrase than the common “historical necessity.” For instance, “My dog is sitting” is true or false no matter what happens in the future, but it is hardly a historical necessity in any useful sense.

12 Were we to engage in extended tense-modal calculations, the Prior notation would be preferable. Since, however, in this essay there are only simple tense-modal calculations, and those few in number, it has seemed better to use a more mnemonic notation.

13 If we were to provide a proof theory, it would be more economical mathematically to have the strong modalities *Sett:*, *Will-always:*, and *Was-always:* as basic.

14 A similar, more complex validity holds in the general case, allowing for first and last moments in *M*; we leave this as an exercise for the reader. Note that Postulate 2 guarantees that any two histories intersect.

15 See Prior [18], 110n3, for the attribution to a multilith by Kamp, UCLA 1967. See also [10].

16 Determinism by definition does not distinguish the actual from the possible (no multiple possibilities). Put determinism to one side now, and confine attention to those philosophers who, like us, take branching to express real possibilities for the future. One might have thought that all such philosophers take actuality as indexical; but this is not the case. Several argue for an objective (non-indexical) view of actuality, for example, Øhrstrøm [15] and Malpass and Wawer [14]. In our judgment their arguments are cogent and interesting, but not, in the end, convincing.

17 You should note that the parameter *m* can be “moved,” for instance, by the tense connectives, that *h* can be “moved,” for instance, by the settledness connective, and that $\delta $ can be “moved,” for instance, by the quantifiers. These parameters are “mobile” in the sense of Section 6B.3 of Belnap et al. [5]. In contrast, neither any connective nor any other operator of the language with which we are dealing can “move” $m_{C}$, which is why it is listed as part of a BH-CIFOL model. Kaplan [11] said that any syntactic construction that “moved” the context-of-utterance parameter would be a “monster,” smuggling in something metalinguistic. There is some debate on whether Kaplan’s ban on monsters is linguistically adequate. Direct quotation does shift context parameters (see, e.g., Belnap [2]), but since it is openly metalinguistic, it should not count as monstrous. There are however more controversial examples (see, e.g., Schlenker [19]). For the purposes of this essay, we simply do not provide any operators that move $m_{C}$.

18 We do not hereby mean to exhaust the natural language uses of “actually;” for an alternative analysis, see Belnap et al. [5], 246f. See also [13].

19 There are certainly unreal possibilities required by epistemic or linguistic considerations. See our discussion in Section 2.

20 This account of at-a-time is awkward at best; it is, however, blessedly brief, while still allowing us to illustrate an important point or two. In particular, one sees that one can make sense of “the open future” without appeal to tenses or other indexical language.

21 Although of course a predicate is a piece of language, whereas a property is an intensional function of a certain kind (Def. 1), our exposition proceeds more smoothly if we let ourselves use “property” sometimes for a closed BH-CIFOL predicate and sometimes for the intension of a closed predicate, whichever is most convenient. With reference to later developments, both “sortal property” and “quality” (or qualitative property) share this harmless ambiguity. A “thing,” in contrast, lives in BH-CIFOL as an individual intension or term falling under the property, *Thing*, defined as in Fact 2 of Section 4.2 below.

22 See, e.g., the discussion in Zanardo [24]. For an author explicitly affirming the “*m* only” option, see, e.g., [7].

23 For discussion, see note 39 of Part I.

24 In the case of infinitely many sortals, we would have instead one axiom each of the form “if it’s a dog, it’s also a thing” to express that belonging to a natural sortal is sufficient for being a thing. There would be no general way to express the corresponding necessity, which in the disjunctive formulation is covered by the biconditional. We may however hope that in a concrete application with infinitely many natural sorts, these would have some hierarchical structure making possible the expression of the necessity.

25 We hereby extend the argumentation given in Belnap et al. [5], 206, which concerned a propositional branching histories framework.

26 Note that this way of setting up things allows our framework to introduce another indexical expression, “person,” or the ethical “we,” to denote the speaker’s kind (or “home form”). In this way, the framework could provide a formal handle for considerations of Aristotelian virtue ethics such as in Thompson [23], in which the “ethical in-group” is determined not via an abstract notion of a person, but by the notion of “one of us” in the broadest sense.

27 It may be more appropriate to render Lewis’s “tomorrow” by an indexical metric tense operator “one day hence” rather than via “at time $t_{1}$.” We stick to our reading because we have not defined metric tenses. The discussion, however, would be exactly analogous.

28 Have you ever heard anybody say such a thing? For what it’s worth, on October 26, 2012, all of Google’s 16 hits for “two futures are equally mine” were direct quotations from Lewis [12]. There was additionally one single hit for “two futures are mine,” also directly linked to Lewis [12]. From the 162.000 results for “two futures,” every other hit that we examined clearly presupposed that the two were incompatible.

29 The early history of Bressan’s book, however, already foreshadowed this regrettable state of affairs. In the sixties, NB, not being well-versed in quantified modal logic, sent the typescript of Bressan’s book to each of two eminent philosophers who did know the topic. One returned the manuscript unread in a week or so; the other kept the manuscript for a year and a half before returning it, still unread. Finally NB, in spite of having doubts as to his background in quantified modal logic, undertook some light editing, the placing of the typescript with a publisher, and the writing of a foreword.

30 See, e.g., [20]. Whether there is a sort of things that exists for a single moment only, seems to be a properly scientific rather than a metaphysical issue. Note, however, that even such momentarily existing things would not be fit to be the extensions in BH-CIFOL, since extensions are relative to cases ($m/h$ pairs), which are more fine-grained than moments.

31 We may venture to speculate that such an investigation should start with the indexically available sortal, human being (“one of us”), see note 26 above; anthropomorphism may then be seen as a result of the fact that the qualities fit for describing human beings were taken to be adequate for all other sorts as well; which, as we know, is not the case. But this had to be learned. For a real episode in the history of science that testifies to the coordination of qualitative and sortal properties, the discovery of platinum comes to mind: A metal identified as platinum was used to make the 1799 standard meter, but a few years later is was discovered that what people had thought to be platinum was in fact an alloy containing various other metals such as osmium and iridium. A more fine-grained handle on qualitative properties allowed for the identification of a new sort.
